# Religion as an influencing factor of right-wing, left-wing and Islamist extremism. Findings of a Swiss youth study

**DOI:** 10.1371/journal.pone.0252851

**Published:** 2021-06-17

**Authors:** Maria Kamenowski, Patrik Manzoni, Sandrine Haymoz, Anna Isenhardt, Cédric Jacot, Dirk Baier

**Affiliations:** 1 Department of Social Work, Institute of Delinquency and Crime Prevention, Zurich University of Applied Sciences, Zurich, Switzerland; 2 School of Social Work Fribourg, University of Applied Sciences and Arts Western Switzerland, Fribourg, Switzerland; Universidad de la Republica, Facultad de Ciencias Sociales, URUGUAY

## Abstract

In criminological research the relationship between religion and delinquency has received great attention. Religiosity has been shown to be a protective factor for violent behaviour, drug use and other types of crime. In contrast, the relationship between religion and extremism was rarely investigated and then almost exclusively in relation to Islamist extremism. This paper presents results of a youth survey on extremism in Switzerland. A total of 8317 young people in ten cantons were interviewed about right-wing, left-wing and Islamist extremism. The study allows in a unique way to analyse religion, religiosity and religious attitudes in relation to three forms of extremist attitudes. The results show that religion is an important influencing factor of extremism, but religious affiliation and religiosity are less important than specific religious attitudes such as religious tolerance and religious exclusivity.

## Introduction

### Religion, religiosity and extremism

#### Previous findings and hypothesis

Criminological theories and research have addressed the relationship of religion and religiosity to deviant behaviour and extreme attitudes, particularly in relation to delinquency. The “hellfire hypothesis” by Hirschi and Stark [1] is considered as the starting point of a plenty of research on the topic of the relationship between religion and delinquency. However, the authors of this hypothesis did not find any significant relationship between religiosity and self-reported delinquency. After their study was published, several other studies reported that religiosity is related to lower self-reported delinquency [for example 2–4] and also deviant behaviour, especially concerning drug use [e.g., 2, 5, 6]. In the past, the relationship between religion and delinquent or deviant behaviour was tested mainly for the Christian faith. It is therefore unclear whether findings regarding Christian faith can be transferred to other affiliations. For example, Baier [7] found for juveniles with an Islamic religious affiliation no significant bivariate correlation between religiosity and violent behaviour; multivariate analyses, however, indicated that Islamic religiosity increases violent behaviour, especially after controlling for alcohol consumption. Religiosity in this study was measured by visiting a place of worship as well as by the frequency of praying and the subjective importance of religiosity in everyday life. Brettfeld [8], on the other hand, reported a similar delinquency reducing effect of religiosity for Christians as well as Muslims regarding violent behaviour. In the case of Muslims, however, the effect was less pronounced and not significant. In this respect, this study also draws attention to the fact that for Islamic religiosity no protective effect comparable to Christian religiosity seems to exist. It can therefore be concluded that religiosity is not a protective factor for violent or delinquent behaviour in general; instead differences regarding religion (e.g., Christian or Muslim) probably exist and should be analysed empirically.

Violent and delinquent behaviour patterns, as well as associated attitudes, can also manifest themselves in various forms of extremism. Regarding the topic of extremism, the influence of individual religiosity was analysed to a much lower degree than delinquency. If one looks at the religious ties, this is examined in particular with Islamist extremism and is interwoven by the dimensions of the attitudes of this form. However, devoted believers do not only exist in the group of Muslims; “one could find devoted believers in many religions who are willing to take extreme measures on behalf of their religious credo” [9]. Fundamentalism is not just a phenomenon among Muslims, but also among Christians, Hindus and so on [cf. for example 10, 11]. Therefore, it is necessary to analyze the relationships between different religions and different forms of extremism. This article focusses on the types of extremism which are relevant for the European Context: Islamist extremism, right-wing extremism and left-wing extremism.

With regard to the social context of Switzerland, it makes sense to examine the influence of two religious’ affiliations: Christianity and Islam. According to public statistics, in 2017 65.6% of the population aged 15 and more belonged to the Christian faith, 5.4% to the Islamic faith; other groups each account for less than one percent of the population (no membership: 26.0%). Regarding to young people, it can be assumed that the proportion of the Muslim population is even higher than in the total population due to demographic trends (immigration from Turkey, former Yugoslavia, Arab countries and a higher birth rate among Muslims) [12]. Comparing these two religious’ affiliations, the present article examines three questions: 1. Does religious affiliation has an influence on three types of extremism? 2. Is the strength of the religious attachment (religiosity) related to the support of extremist attitudes? 3. Do other religious attitudes influence the acceptance of any form of extremism? Regarding these other attitudes, the influence of religious exclusivity as well as of religious tolerance are considered.

*Findings on religion and Islamist extremism*. Especially in relation to Islamist extremism it is discussed whether religiosity represents a risk factor of radicalisation. The empirical findings are contradictory. A qualitative study among prison inmates by Aslan, Akkılıç and Hämmerle [13] supported the perspective that Islamic religiosity is one of the reasons for turning to Islamist extremism. Also, Beller and Kröger [14] reported that a more frequent religious practice (visiting mosques) went hand in hand with a stronger support of extremist violence. However, their results also showed that the personal significance of religion lowers the support of extremist attitudes (see e.g. also [15]. The authors’ analysis thus emphasized that a differentiated operationalization of religiosity is necessary and can possibly lead to different correlations [14], although due to the strong overlap between religious practice and personal significance of religion a separated analysis of these indicators can be questioned. In addition, it should be kept in mind that extremists are sometimes religious illiterates or converts who were not influenced by Islamic religious socialization [16]; thus, they may not be fully familiar with Islam, but they can still be strongly religious. These findings indicate that different correlations between Islamic religion and Islamist extremism may depend on the operationalisation of religiosity. Furthermore, it can be concluded that Islamic religiosity does not seem to be a strong preventive factor of Islamist extremism.

*Findings on religion and Right-wing extremism*. Although the connections between Christian religion and right-wing extremism have certainly gained attention, yet only few empirical studies exist on this topic [17, 18]. After the Second World War, the “Christian Occident” became a central motif of right-wing nationalist and conservatives in Europe, which had a national identity-forming effect. And still today the movements and parties such as the “Alternative for Germany” still see themselves as defenders of the Christian Occident and Christian culture. Here, religion is used to mark the difference to other religions, especially the Islam (see [17, 19, 20]. The fact that the radical right takes up the subject can be explained rather as a reaction to the pluralization of society than to the actual turn of people towards religious faith [18]. In the case of right-wing extremism, the criteria for exclusion can be justified ethnically, but also culturally and thus also religiously. Hoyningen-Huene [21] distinguishes between three types of right-wing extremist youth: traditionally right-conservative youth, evangelical-fundamentalistic youth and youth who have been socialized in a Christian church. This typology illustrates that there is an overlap between Christian religiosity and right-wing extremism. This conclusion is also supported by an analysis of song lyrics of bands classified as right-wing music groups [21, 22]. Hoyning-Huene [21] identifies even eight groups showing relationships between new religious and pagan movements and right-wing extremism (cf. [23]). Pauwels and De Waele [24] have investigated the relationship between religious authoritarianism and right-wing extremism. They found that especially for Christian fundamentalism there is a link with right-wing extremist attitudes (and also violence).

*Findings on religiosity and Left-wing-extremism*. Left-wing extremism is defined as a political orientation whose ideological goal is—among others—the establishment of communism or anarchy. A characteristic of this thinking is the rejection of any religious views and leaders (a-religiosity). This suggests that there should be no direct relationship between religion or religiosity and left-wing extremism. Looking at the literature, nearly no empirical study on the relationship between religiosity and left-wing extremism is available. Deutz-Schroeder and Schroeder [25], based on a representative survey of the German population, diagnosed that left-wing extremists are less likely to belong to a religious community. Ramiro [26] finds comparable results for several Western European countries. Left-wing extremist groups, however, are characterised by a great diversity, so perhaps religious-socialist orientations exist (e.g., liberation theologians), but religiosity is typically not the most characteristic factor about left-wing extremism. In the following, the relationship between these factors will therefore be examined in an exploratory manner.

#### Hypotheses

Religion has different dimensions and this paper looks at three of them: belonging to a religious group (affiliation), the strength of religious bonding (religiosity), and religious attitudes (e.g. about exclusivity and tolerance). Regarding affiliation, a distinction is made between three religious’ groups in this article due to the situation in Switzerland: Christians (Catholics, Protestants, Protestant Free Churches, Orthodox), Muslims (e.g. Sunnis, Shiites, Alevis) and persons without religious affiliation. Right-wing, left-wing and Islamist extremism will be analysed as types of extremism, with attitudes, not behaviour, being examined in each case.

Regarding religious affiliation, it is expected that an affiliation alone does not yet have a formative effect on attitudes and behaviour. In Western societies, membership is becoming less and less important, i.e., people sometimes belong to religious groups, but do not practice faith. At the same time, people who do not belong to a religious community often show a certain degree of faith. Thus, it can be assumed that the degree of religiosity is related to extremist attitudes, not affiliation per se:

Hypothesis H_1_: Persons affiliated to a religion do not agree more frequently with extremist attitudes than persons without religious affiliation; however, the degree of religiosity is correlated with extremist attitudes.

Considering the research results reported above, it can be assumed that Muslim religiosity may be a risk factor for Islamist extremism. Christian religiosity, on the other hand, should predominantly have a protective effect regarding extremist attitudes, whereby with regard to right-wing extremist attitudes it can also be assumed that a strong Christian religiosity correlates with a higher approval:

Hypothesis H_2_: A stronger Christian religiosity reduces agreement with left-wing extremist and Islamist extremist attitudes; on the other hand, the more religious Christian respondents are, the more likely they agree with right-wing extremist attitudes.
Hypothesis H_3_: The stronger the Muslim religiosity, the higher is the agreement with Islamist extremist attitude.

If it is assumed that religiosity correlates with a higher approval of extremist attitudes, then this should be the case not solely due to religious feelings and orientations, but to a certain interpretation of a religion. If religiosity is accompanied by humanistic, universalist values that are at the core of every world religion, then a violence and extremism reducing effect should be found for every form of religiosity. If, on the other hand, stronger religiosity is accompanied by intolerance towards other religions and claims of exclusivity in relation to one’s own religion, then negative consequences of religiosity are to be expected. There are some empirical studies showing an association between religious behaviour and prejudice, demonstrating that religious affiliation and religiosity as such are not the problem, but that religious views serve the individual to provide orientation and (identity-) stability (see already [27, 28]). Religiosity as well as prejudice can be a useful means for gaining a stable identity. In line with that argument, Hyoningen-Huene [21] noted that the concept of the exclusivity of one’s own religion includes “the claim to be in possession of the valid truth […] In a radical consequence this leads to an unquestionable claim to truth and thus intolerance towards other religions and worldviews”. However, the question of which kind of relationship, if any, exists between religious intolerance and exclusivity and extremism still waits for an answer. Keeping in mind that radicalization also involves an exclusionary attitude and the conviction of being in possession of a certain and only truth, these all could be based on religion [29]. Thus, extremist attitudes and religious exclusivity and intolerance may reinforce each other:

Hypothesis H_4_: Religious attitudes like intolerance and claims of exclusivity increase the agreement with extremist attitudes.

If only religion-related variables are considered in the analyses, the influence of religion on extremist attitudes may be overestimated. For this reason, we consider several control variables with evidence of influencing both extremist attitudes and religiosity.

Looking first at socio-demographic variables, *gender* should be included in the analyses. Numerous studies have reported correlations between male gender and right-wing extremism. This finding is interpreted as a socialisation effect [30]. However, there are mixed findings regarding gender differences in left-wing extremism. Schroeder and Deutz-Schroeder [31] found no differences in all dimensions of left-wing extremism they analysed, while Baier and Pfeiffer (2011) [32] demonstrated that boys agreed stronger with left-wing extremist attitudes than girls. In relation to Islamist extremism, e.g. Eser Davolio et al. [33] showed that most jihadists (i.e., travellers from Switzerland to the Jihad in Irak or Syria) are men. However, Eser Daviolio and Lenzo [34] also note that women play a relevant role, but mostly one that is less perceived by the public and usually takes other forms of support than fighting (cf. also [35]).

Another control variable is *migration background*. The assumption here is that more young people without a migration background favour right-wing extremist attitudes; on the other hand, people with a migration background are more likely to accept Islamist extremist attitudes if they are muslims, e.g. because of their affiliation to the Islam.

Additionally, a higher *education* is classified as a protective factor especially for right-wing extremism [36, 37]. For left-wing extremism, Baier and Pfeiffer [32] showed the same effect: Students who attend a lower type of school have higher levels of extremism than students who attend a higher type of school. Also, for Islamist extremism there is empirical evidence that a high education is a protective factor (e.g. [38]).

Beside this, it is assumed that right-wing extremism is a result of the threat to personal prosperity, to so-called *deprivation experiences* (e.g. [39, 40]). People with a low social status and stronger experiences of deprivation therefore agree more frequently with right-wing extremist attitudes. No research results are available on the influence of deprivation on left-wing extremism. Regarding Islamist extremism, it is also assumed that deprivation can have a negative impact. For instance, Böckler [41] concluded that feelings of relative deprivation cause young people to turn to (Islamist) extremism. El-Mafaalani [42] also stated that disadvantaged young people with experiences of exclusion are more likely to become Islamist extremists.

Beyond demographic variables and deprivation, the influence of different socialisation and personality factors should be taken into account. First, it can be assumed that *parenting styles* play a role. According to Baumrind [43], two dimensions are particularly important: parental attention and parental control. Parental control, the dimension that is considered in the empirical analyses below, is about parents knowing about their children’s activities, whereabouts, friends, etc.; this knowledge enables them to detect and sanction misconduct. Previous empirical research has shown that children of controlling parents are more likely to be protected from becoming delinquent (e.g. [44–46]); the same is expected for all types of extremism.

Second, *self-control* can be classified as a relevant influencing factor of extremism [24]–and higher level of self-control is also associated with greater religiosity [47]. High self-control is perceived as a protective factor, whereas a person with low self-control is not able to resist current needs, prefers simple tasks as well as risk-taking behaviour and is impulsive and self-centred [48] and therefore more prone to engage in extremism. Pauwels and De Waele [24], for example, found that political violence is related to lower levels of self-control.

Third, *moral values* may have an impact on extremist attitudes. According to Wikström [49], crime propensity depends on two conditions: the moral values of an individual and the ability of self-control. The importance of these moral values for explaining delinquent behaviour was confirmed, for example, by Schepers and Reinecke [50]. Pauwels and Schilds [51] assume that a relationship should also exist with extremism.

Fourth, as with almost all forms of deviance in adolescence, the peer group has to be considered. A lot of studies showed that having contact with delinquent peers enhances own delinquent behaviour (e.g. [52–54]). An extremist radicalisation is also a group process that can be described as de-individuation, shift from individual normative standards to group standards, social identity formation, polarisation and so on (see e.g. [55]). With regard to their friendship networks, it should be noted that young people in particular are heavily dependent on what is available in their immediate environment; they do not consciously select specific extremist groups to which they would like to belong, but rather fall into extremist right-wing, left-wing or Islamist extremist groups as a result of what is in their environment (cf. e.g. [56, 57]). If the considerations on the control variables are summarised, the following can be assumed:

Hypothesis H_5_: Socio-demographic variables as well as deprivation/low social status, low parental control, low self-control, low moral values and friendships with delinquent peers are risk factors of extremist attitudes; however, the influence of religiosity and religious attitudes is still found when controlling for these factors.

## Sample and procedure

### Sample

A sample of young people living in Switzerland is used to test the hypotheses. The data were collected within the study “Political Extremism among Young People in Switzerland: Distribution and Influencing Factors”, which was founded by the Swiss National Science Foundation (contribution number 100017_165760). The Cantonal Ethics Committee of the Canton of Zurich has determined that the study is ethically unproblematic and does not fall under the Human Research Act. The aim of the study was to determine the prevalence and influencing factors of various forms of extremism in Switzerland. In order to obtain the sample, the following procedure was followed: The survey did not claim to be representative for whole Switzerland, as this would have been very difficult to achieve for a total of 26 cantons. Instead, the survey was conducted in ten cantons: Basel-Land, Bern, Fribourg, Geneva, Lucerne, Solothurn, St. Gallen, Ticino, Valais and Zurich (representing both rural and urban regions, as well as the three dominant language regions). The survey was aimed at young people aged between 17 and 18 years on average, because it could be assumed that extremist attitudes have a higher prevalence in this group in comparison to younger adolescents. Accordingly, all types of schools in the ten cantons in which young people of this age are taught were included in the random sampling (vocational school, transitional education, high school (gymnasium) and technical/commercial secondary school). In the cantons, a random drawing of schools or school classes was then carried out, in which surveys were to be conducted.

Students were interviewed with an online questionnaire during one school lesson (45 minutes); the interviews were administered by trained interviewers or teachers. During the survey, a class work atmosphere was created, i.e. the pupils were, for example, set apart and it was ensured that they completed the questionnaire in a disciplined manner. Anonymity and confidentiality were guaranteed. The survey was voluntary, and the young people had the opportunity to decide for themselves whether to participate in the survey or to stop it at any time. The respondents were informed about this in the classes and in the primary text of the survey. The parents of the students were informed with a letter before the survey. The parents’ consent to the child’s survey was not obtained, as the young people were able to decide for themselves to take part, given their age. However, the parents had the opportunity to indicate that they did not want their child to participate in the survey.

In total, 595 classes with all in all 8317 students took part in the survey. The total response rate of the survey was 39.1%. This is a relatively low rate because many schools refused to take part in the survey. If schools and classes agreed to participate, nine out of ten pupils took part in the survey.

With 55.8% the majority of the students are 17 or 18 years old; 22.5% are younger. The gender ratio of the survey is balanced (male youths 49.7%, female youths 50.3%). Of all respondents, 52.0% attend vocational school, 12.3% technical/commercial secondary school, 26.4% high school (gymnasium) and 9.3% transitional education–the last type of school is attended by those young people who were unable to start vocational training after completing school. Less than half of the students (47.9%) do not have a migration background (that is, no parent was born outside of Switzerland). Therefore, the proportion of young people with a migration background is quite high with 52.1%, which is not surprising given the fact that Switzerland is an immigration country.

### Instruments

#### Extremism scales

In this article, three types of extremism are analysed: right-wing extremism, left-wing extremism and Islamist extremism. The items used for measuring these types of extremism can be found in S1–S3 Tables (which base in part on the instruments of e.g. [29, 32, 58, 59]).

*Right-wing extremism* was defined as an orientation that advocates nationalism and divides people into groups of different values (social darwinism), whereby the members of the own group are whites (racism) and the members of the foreign group are mainly foreigners, Muslims and Jews, among others, which is why xenophobia, Islamophobia and antisemitism represent further core dimensions of right-wing attitudes. Extremism, however, is not only characterized by the agreement to certain ideological goals, but also by a willingness to carry out violence against groups of persons defined as enemy groups, which is why the willingness to exercise verbal or physical violence against foreigners and left-wing extremists was also measured. In total, the scale for measuring right-wing extremist attitudes comprises ten items, which could be agreed with from “1—not true at all” to “6 –completely true”. The reliability of the scale amounts to Cronbach’s Alpha = .88; the selectivity of the items is at least.44. As the mean values of the individual items presented in S1–S3 Tables show, the items are only approved of to a small extent. The items were combined into a mean scale (the same applies to the other extremism scales).

*Left-wing extremism* was defined as an orientation whose ideological goal is the establishment of communism or anarchy. The enemies of this orientation are on the one hand the capitalist economy (hostility towards capitalism), on the other hand the state and its organs classified as repressive, especially the police (hostility towards police and state). Again, additionally the willingness to carry out violence against groups of persons defined as enemy groups was integrated in the instrument. Because of that there are four items measuring the willingness to exercise physical violence against capitalists, police officers and right-wing extremists. The left-wing attitudes scale comprises of nine items, which could be agreed with from “1—not true at all” to “6 –completely true”. The reliability of the scale amounts to Cronbach’s Alpha = .79; the selectivity of the items is at least.33. The approval of the items is sometimes significantly higher than the approval of the right-wing extremism items.

The main ideological goal of *Islamist extremism* is the introduction of a theocracy based on the Koran and Sharia. The own group of orthodox Muslims is upvalued (superiority of Islam); the West in general and non-traditional Muslims are regarded as enemy groups (devaluation of western societies, hostility towards non-traditional Muslims). In addition, inhabitants of the western country in which the Muslims live are classified as enemies, in Switzerland accordingly the Swiss (hostility towards Swiss). The willingness to use violence is directed against non-Muslims on the one hand. On the other hand, terrorist attacks are a central mean of achieving the goals of Islamist extremism. In order to be able to use violence against groups declared as enemies, a trip to war zones is sometimes necessary, which is why the advocacy of so-called jihad journeys also represent a facet of the readiness to use violence of Islamist extremism (advocacy of terrorism/IS). The Islamist extremism attitudes scale comprises of eleven items, which could be agreed with from “1—not true at all” to “6 –completely true”. The reliability of the scale amounts to Cronbach’s Alpha = .81; the selectivity of the items is at least.36. The approval of the items is rather low.

The means of the three scales are 1.85 (right-wing extremism), 2.20 (left-wing extremism) and 1.56 (Islamist extremism), showing that left-wing extremism enjoys a higher level of approval among Swiss youths than right-wing and Islamist extremism. All scales are right-skewed (high values, i.e. agreement, are found less frequently than low values, i.e. disagreement), with skewness ranging from 0.995 to 1.609 (standard error:.028). For this reason, a robust estimator is used in the following analyses (MLR).

#### Religious affiliation, religiosity and religious attitudes

In order to ascertain *religious affiliation*, the young people were asked to which religious community they belong. Here it was possible to report a variety of different types of religious affiliations. Four groups were distinguished for the analyses: Respondents with Christian affiliation (majority Catholic, but also Protestants, Protestant Free Churches, Orthodox), respondents with Muslim affiliation (majority Sunnis), respondents with other affiliations and respondents without religious affiliation. The sample comprises of 4814 Christians (59.6% of sample), 776 Muslims (9.6%), 369 students with other affiliation and 2122 students without an affiliation (236 missing cases).

To measure *religiosity*, the following five items of an instrument developed by Huber [60] were used: 1. How often did you think about religious topics in the last 12 months? 2. How often did you attend church services, fellowship prayers or the like in the past 12 months? 3. How often did you pray in the last 12 months? 4. How often did you experience situations in the last 12 months in which you had the feeling that God or something divine intervened in your life? 5. I believe that there is God or something divine. The answer categories of the first four items ranged from “1—never” to “7—daily”, the answer categories of the fifth item from “1—not true at all” to “6—completely true”. Every single item stands for one dimension of religiosity distinguished by Huber (2008) [58]; these are intellect, public practice, private practice, experience and ideology. The wording of the items has been changed in part compared to the original instrument. To determine an individual value of religiosity, in a first step the response categories were transformed to the same range from 1 to 7. In a second step, the reliability of the instrument was checked for the religious groups mentioned. For Christians Cronbach’s alpha is.84, for Muslims.77 (other affiliation:.83). In the third step, the mean value of all five items was calculated, considering only those respondents who stated that they belonged to a religious group. Table 1 presents the means of the single items and the scale separately for the two religious’ groups (answer categories of the fifth item transformed to the seven-point response scale). On the one hand, in both groups the first and especially the fifth item receive a particularly high approval. On the other hand, for each item and accordingly also for the scale there is a higher mean for Muslim students compared to Christian students.

**Table 1:** Descriptive statistics of the religiosity and religious attitudes scales for Christians and Muslims.

Variable	Item	Christians		Muslims	
		mean	Std. dev.	mean	Std. dev.
**religiosity**	How often have you thought about religious topics in the last 12 months?	3.09	1.65	4.19	1.96
	How often have you attended church services, fellowship prayers or the like in the past 12 months?	2.33	1.18	2.57	1.76
	How often have you prayed in the last 12 months?	2.88	1.89	3.31	2.21
	How often have you experienced situations in the last 12 months in which you had the feeling that God or something divine intervened in your life?	2.33	1.65	3.84	2.19
	I believe that there is God or something divine.	4.81	1.98	6.15	1.48
	**scale**	**3.08**	**1.32**	**4.00**	**1.41**
**religious exclusivity**	I am convinced that my religion is right on religious issues.	2.98	1.59	4.25	1.60
	I am convinced that other religions are wrong about religious issues.	2.21	1.35	2.73	1.59
	My religion is the only true religion.	2.07	1.50	3.53	1.99
	**scale**	**2.41**	**1.24**	**3.49**	**1.45**
**religious tolerance**	For me, every religion has a true core.	4.21	1.50	4.54	1.45
	I think one should be open to all religions.	4.75	1.43	5.09	1.27
	**scale**	**4.49**	**1.25**	**4.81**	**1.12**

Additionally, two types of religious attitudes were measured by calculating mean value scales: claim for *religious exclusivity* and *religious tolerance*. The claim for exclusivity was measured with three items (see Table 1). Cronbach’s Alpha for Christian students was.79, for Muslim students.78. Religious tolerance was measured using two items (Table 1). The two items correlate r = .47 for Christians and r = .38 for Muslims. If the means of the mean scales are considered, Muslim youths show a higher approval of exclusivity. Regarding religious tolerance, the differences between the two groups is less pronounced, but Muslim students show a higher mean than Christian students as well. Religious exclusivity and religious tolerance correlate to r = -.23 for Christians and to r = -.15 for Muslims with each other.

#### Control variables

*Gender* of the student was recorded with the dichotomous variable female (0) and male (1). The *migration background* was also measured dichotomously (0—both parents were born in Switzerland, 1—at least one parent was born abroad). With regard to the *education* of the respondents, a distinction is made between respondents with a high level of education (1, attending high school) and respondents with a medium or low level of education (0). *Deprivation resp*. *a low social status* (1) is indicated by the fact that respondents or their parents receive social assistance or that at least one parent is currently unemployed. If there is no receipt of social assistance and no parent is unemployed, a higher status (0) is assumed.

Beside these socio-demographic variables, *parental control* was measured based on the considerations of Baumrind [43]: Students had to rate how often their parents “knew where I am when I was not at home”, “knew what I do when I was not at home” and “knew which friends I am with when I was not at home” when they were younger. The answer scale ranged from “1—never” to “5—very often”. Cronbach’s alpha of the scale is.85, thus a mean scale was calculated.

*Self-control* was measured in the current study using nine items depicting impulsiveness, risk-seeking and self-centeredness of a person [61] which could be agreed with from “1—not true at all” to “6—completely true”. Examples are: “Sometimes I will take a risk just for the fun of it.” or “I try to look out for myself first, even if it means making things difficult for other people.” The reliability of the mean value scale is Cronbach’s alpha.82.

*Moral values* were asked in relation to the legal system (see [62]). They were measured using five items which were summarized to a mean value scale: introduced by the question “How wrong do you think it is when someone your age does the following?” On a scale ranging from “1 –not wrong” to “4 –very wrong” students had to judge these behaviours: 1. Intentionally insulting someone because of his religion, colour or national origin; 2. Intentionally damage/destroy property that does not belong to him/her; 3. Breaking into a building to steal something; 4. Hit someone to hurt the person; 5. Use a weapon or violence to get money or other things from other people. The reliability of the scale is very good (Cronbach’s Alpha = .93).

To measure contact with *delinquent peers*, students were asked how many friends they have who have done the following in the last 12 months ([63]): stole something in a shop, broke into a building to steal something, taken something from someone by force, beat and hurt another person, taken drugs (hashish, ecstasy, etc.). The answer categories ranged from “1 –zero friends” to “6—over 10 friends”. A maximum value scale was calculated; i.e. the highest number of reported delinquent friends was coded in the scale (Cronbach’s Alpha = .80).

All means and standard deviations for the control variables for the total sample are shown in Table 2. Due to the different response categories, the means cannot be directly compared. However, it can be concluded that the students have experienced a high degree of parental control, have high rather than low self-control and strongly agree with moral values.

**Table 2:** Descriptive statistics of the control variables.

	Variable	mean	Std. dev.
**Socio-demographics**	sex: male	0.50	0.50
	migration background	0.52	0.50
	education: high	0.26	0.44
	receipt of unemployment benefit/welfare	0.16	0.36
**Socialisation and personality**	high parental control	4.07	0.88
	low self-control	2.97	0.88
	high moral values	3.56	0.73
	Number of delinquent peers	2.77	1.83

## Results

### Religious affiliation and extremism

In order to test the first and the fifth hypothesis, multilevel linear regressions were calculated (because students were nested in classes) in which religious affiliation were introduced as dummy variables; Mplus 7.31 and the MLR estimator were used for the analyses. According to the hypothesis, the group of respondents without a religious affiliation are the reference category. In the first regressions, only the religious affiliation was considered, in the second regressions additionally the control variables. The explained variances of the first models of right-wing and left-wing extremism is very low—so religious affiliation actually has only a low explanatory value. Due to the high number of cases, however, the standardised coefficients are repeatedly shown to be significant, even though their magnitude can be classified as low.

Christian respondents agree more frequently with right-wing extremist attitudes than respondents without an affiliation. Results also show that Christian respondents are less likely to agree with left-wing extremist attitudes. However, this effect is reduced after consideration of the control variables. There are also findings that Christian respondents agree more frequently with Islamist extremism than respondents without an affiliation. The findings remain stable even after consideration of the control variables.

With regard to the explanation of Islamist extremism, the effect of religious affiliation is stronger than in the other forms of extremism. The explained variance of the first model is 8.9%, this mainly because of the strong effect of the Muslim group: Compared to respondents without an affiliation, they much more often agree with Islamist extremist attitudes (.32). Muslim respondents also more often agree with both right-wing and left-wing extremist attitudes—but the effect is very small.

Looking at the control variables, the results show that male respondents agreed significantly more with extremist attitudes than female respondents, with the strongest effect in relation to right-wing extremist attitudes. In particular, students with a migration background agree less with right-wing extremist attitudes than students without a migration background. A high level of education is a protective factor—especially with regard to right-wing extremism and Islamist extremism. A low social status as well as parental control only have a small effect on extremist attitudes (standardised coefficients consistently under.10). A low level of self-control increases all three forms of extremism to roughly the same extent; high moral values on the other hand reduce all extremist attitudes. Correlations with delinquent peers are rather weak and contradictory: The more delinquent friends respondents have, the less they agree with right-wing extremist and Islamist extremist attitudes; on the other hand, there is a positive correlation with left-wing extremist attitudes.

### Religiosity and extremism

Hypotheses 2 and 3 were tested using bivariate correlations–again controlled for the multilevel data structure. The results are presented in Table 3 (for multivariate findings see Table 4). The stronger the religiosity among Christian youths, the more likely they are to agree with left-wing and Islamist extremist attitudes–but the correlations are very small. For right-wing extremism, the correlation with Christian religiosity is not significant. A stronger Muslim religiosity is accompanied by a stronger agreement with left-wing extremism and especially for Islamist extremism (.12 resp..31). Again, no significant correlation between right-wing extremism and Muslim religiosity is found.

**Table 3:** Correlations between religiosity and extremism attitude scales (standardised correlations shown; estimator MLR).

	Right-wing extremism	Left-wing extremism	Islamist extremism	N students/N classes
Christians: religiosity	-.02	.06***	.05**	4561/581
Muslims: religiosity	.09	.12**	.31***	689/320

* p < .05,

** p < .01,

*** p < .001

**Table 4:** Multilevel linear regression of religiosity and religious attitudes on extremism attitude scales (standardised coefficients shown; estimator MLR).

	Right-wing extremism		Left-wing extremism		Islamist extremism	
	Christians	Muslims	Christians	Muslims	Christians	Muslims
religiosity	-.09***	-.03	-.01	.01	-.05**	.05
religious exclusivity	.34***	.24***	.17***	.22***	.28***	.54***
religious tolerance	-.27***	-.08	-.01	-.05	-.09***	-.19***
sex: male	.07***	.11**	.12***	.17***	.02	.05
migration background	-.13***	-.06	.07***	.00	.06***	-.01
education: high	-.11***	-.12***	-.06***	-.06	-.14***	-.06*
receipt of unemployment benefit/welfare	.01	-.03	.04*	.02	.05***	.02
high parental control	.03*	-.11**	-.07***	-.04	-.04*	-.01
low self-control	.19***	.13**	.20***	.21***	.18***	.14***
high moral values	-.12***	-.25***	-.10***	-.09*	-.17***	-.14**
Number of delinquent peers	-.06***	.00	.08***	.11**	-.06***	.01
**R**^**2**^	**0.397**	**0.270**	**0.193**	**0.238**	**0.260**	**0.481**
**N students**	**4095**	**607**	**4095**	**607**	**4095**	**607**
**N classes**	**576**	**302**	**576**	**302**	**576**	**302**

* p < .05,

** p < .01,

*** p < .001

### Religious attitudes and extremism

To test hypothesis 4, again bivariate correlations were calculated, separately for the two religious’ groups of Christian and Muslim students. The results are identical for both religious groups: the stronger the attitudes of religious exclusivity are, the higher the agreement with all extremist attitudes is. There is a strong correlation between exclusivity and Islamist extremism among Muslim youths (.61), but exclusivity also correlates closely with right-wing extremism among Christian youths (r = .41). The stronger the tolerance, the lower the approval of extremist attitudes—this applies to all forms of extremism (with lower correlations for left-wing extremism) and to both religious groups (Islamist extremism among Muslim youths -.32 and right-wing extremism among Christian youths -.44; see Table 5).

**Table 5:** Correlations between religious exclusivity/religious tolerance and extremism attitude scales (standardised correlations shown; estimator MLR).

	Right-wing extremism	Left-wing extremism	Islamist extremism	N students/N classes
**religious exclusivity**				
Christians	.41***	.22***	.33***	4459/581
Muslims	.28***	.28***	.61***	665/316
**religious tolerance**				
Christians	-.44***	-.11***	-.23***	4263/580
Muslims	-.22***	-.17***	-.32***	640/309

* p < .05,

** p < .01,

*** p < .001

The various religiosity variables were finally included in multilevel linear regressions, whereby the control variables were again integrated. The results are presented in Table 4. These show that the religiosity of a respondent has largely no effect on extremist attitudes. In case of Christian students, it is found that a stronger religiosity lowers right-wing extremist and Islamist extremist attitudes significantly—but the standardised coefficients are very small (-.09 and -.05). In contrast, the attitude of religious exclusivity shows a significant and strong correlation for all groups with all extremist attitudes. The more exclusivity claims are expressed, the higher the agreement with extremist attitudes is. At the same time, religious tolerance does not lower extremist attitudes to the same extent: Stronger correlations can only be observed for Christian youths and right-wing extremist attitudes and for Muslim youths and Islamist extremist attitudes.

The results on the control variables largely correspond to the effects already reported in Table 6: Male gender is a risk factors for extremist attitudes with smaller effects of gender on Islamist extremism. Respondents with a migration background less likely agree with right-wing extremist attitudes, but more likely with left-wing extremist or Islamist extremist attitudes—at least when they are Christians (but the effects are again rather small). A higher education is a protective factor—but all in all the influence of education is rather small and sometimes not significant. Low social status is largely uncorrelated with extremism. Parental control is largely of weak influence, with some correlations showing that high parental control goes hand in hand with lower extremism. Low self-control correlates significantly with all extremist attitudes. Moral values, on the other hand, are an important protective factor. Acquaintance with delinquent friends lowers acceptance of right-wing extremism and Islamist extremist attitudes, while at the same time increasing acceptance of left-wing extremist attitudes.

**Table 6:** Multilevel linear regression of religious affiliation on extremism attitude scales (standardised coefficients shown; estimator MLR).

	Right-wing extremism		Left-wing extremism		Islamist extremism	
no religious affiliation	reference	Reference	reference	reference	reference	reference
Christian	.10***	.10***	-.08***	-.03*	.10***	.10***
Muslim	.01	.04**	.04*	.05**	.32***	.29***
other	.02	.04**	.04**	.04**	.11***	.09***
sex: male		.12***		.10***		.04***
migration background		-.14***		.04***		.05***
education: high		-.19***		-.07***		-.16***
receipt of unemployment benefit/welfare		.00		.04**		.04**
high parental control		.02		-.07***		-.02
low self-control		.22***		.22***		. 20***
high moral values		-.20***		-.09***		-.19***
Number of delinquent peers		-.10***		.12***		-.07***
**R**^**2**^	**0.009**	**0.193**	**0.013**	**0.171**	**0.089**	**0.230**
**N students**	**7370**	**7370**	**7370**	**7370**	**7370**	**7370**
**N classes**	**594**	**594**	**594**	**594**	**594**	**594**

* p < .05,

** p < .01,

*** p < .001

## Discussion

This study analysed the influence of religion and related attitudes on extremist attitudes. It is striking that it looks at three different forms of extremism simultaneously and examines various indicators of religion (affiliation, religiosity, religious attitudes) with regard to their influence, with a focus on comparing Christian and Muslim youth. In the following, the findings are summarized along the hypotheses and conclusions are drawn.

The analysis showed that religious affiliation explains extremist attitudes in different ways. Belonging to a Christian religious affiliation is associated with a slightly higher acceptance of right-wing extremist as well as Islamist-extremist attitudes. However, it has been shown that belonging to Islam is accompanied by an approval of Islamist extremism. As a result, the first part of hypothesis 1 is not supported by the data. Nevertheless, an affiliation seems to be important for extremist attitudes. The religious socialisation of young people (both in family and in wider social context) should require a critical examination of the religious content conveyed, because it is deliberately used by extremist groups.

The second part of the first hypothesis is also partially not confirmed: The degree of religiosity correlates with extremist attitudes in Christian youth at negligible levels. Only for Muslim youths it is found that a more pronounced religiosity strengthens left-wing extremist, but especially Islamist extremist attitudes. Thus, when religiosity is considered, the result for Muslim youths is: The more religious they are, the more important extremist attitudes are to them. This corresponds completely to the formulated hypothesis 3. Hypothesis 2, however, is not confirmed: Among Christian youths, a more pronounced religiosity does not substantially reduce the approval of left-wing and Islamist extremist attitudes. In addition, the bivariate analysis shows no correlation with right-wing extremist attitudes. Christian religiosity is thus not related to extremist attitudes. This also means that it is not a protective factor of such attitudes in this study.

Hypothesis 4 is largely confirmed by the analyses presented. The claim of religious exclusivity increases the agreement with extremist attitudes among both Christians and Muslims. And religious intolerance, which was operationalized in the analyses by means of a short scale for measuring religious tolerance, is also accompanied by a higher agreement with extremist attitudes among both Christians and Muslims. Further multivariate analyses have revealed important additional findings: Firstly, religious exclusivity in particular proves to be an influencing factor of extremist attitudes—irrespective of the religious affiliation. At the same time, the strongest correlation results for Muslims and Islamist extremist attitudes; i.e. if Muslims claim religious exclusivity, then the support for Islamist extremism is particularly strong. For prevention work this means that this particular form of religious conviction must be countered. Those who believe that their own religious group is something special tend to see extremism as an opportunity to assert their own group interests. Extremisms thus apparently promise young people who, for whatever reason, have a high claim of exclusivity a possibility to live out this claim.

In relation to these attitudes it is conceivable, however, that further factors should be considered in further studies, which are influential in the background (e.g., rigidity, authoritarianism, thinking in black and white terms) show.

The claim to exclusivity is more important for extremist attitudes than religious intolerance. Religious intolerance, for example, does not influence left-wing extremist attitudes; and the influence on Islamist extremism is also rather weak.

The findings of the multivariate models show that, when religious exclusivity is included in the analyses (together with control variables), the strong effect of religiosity on Islamist extremist attitudes among Muslim youths completely disappears. The problem is therefore not a strong belief in Islam, but rather if this belief is linked with claims of exclusivity in the course of religious socialization. Here a critical discussion within the religion about this claim for exclusivity seems of special importance.

All reported findings are largely independent of the additionally included control variables; in this respect, hypothesis 5 is confirmed. At the same time, the findings on the control variables themselves offer further interesting results that need to be discussed (and examined in future analyses). For example, (financial) social status is not an important factor influencing extremist attitudes, so extremism is not particularly attractive for socially disadvantaged young people. Moreover, it is found that parental control and also migration background correlate only weak with extremist attitudes. As far as parental control is concerned, this may be due to the fact that older youths have been analysed who have probably largely detached themselves from the parental home and its control. The level of juveniles’ education can be classified as a protective factor of extremist attitudes, although here, too, the influence of higher education is all in all rather weak. Therefore, extremist attitudes are also partly attractive for higher educated young people. Male adolescents are more open to extremist attitudes; although the correlations are also weak and, in some cases, not significant, which is certainly related to the fact that variables such as self-control or moral values have been taken into account that partly explain gender effects.

Contrary to the criminological literature, there is no strong connection between the association with delinquent friends and extremism. This may be explained by the fact that attitudes, not behaviour, were considered. For right-wing extremism and Islamist extremism, having delinquent friends even seems to be a protective factor. However, since the corresponding coefficients are low (and only exist among Christian youths), we will refrain from interpreting them here.

Central influencing factors of all extremist attitudes are low self-control and low morality. The findings thus also are in line with assumptions of the theory of self-control.

The study has a strength in analysing religion and extremist attitudes in a differentiated manner and could also draw on a large sample of Christian and Muslim youth. Nevertheless, it has a number of limitations that should be mentioned at the end. For example, it concentrates only on young people. A comparison with older age groups would undoubtedly be desirable. Moreover, it is only a cross-sectional study, which does not permit any conclusive statements about cause-and-effect relationships. The response rate of the survey is below average for school-class based surveys, so that it cannot be ruled out that the groups ultimately reached are in some way selective. In addition, all data are based on self-reports; the ratings on religiosity or extremist attitudes cannot be validated by other sources. Ultimately, the analyses only examined attitudes for which it is unclear whether they can explain behaviour. The findings cannot therefore be applied, for example, to the explanation of terrorist behaviour. Although the sample was large, it does not allow for differentiation of subgroups within the religious groups (e.g. Catholics and Protestants); young people of other religions could not be considered in detail in the analyses either. With regard to the measuring instruments used, it should be mentioned as a limitation that, firstly, extremist attitudes were surveyed with partly newly developed instruments, which are to be validated in further studies. Secondly, measurement of religiosity focuses largely on traditional religiousness. Forms of religiosity that deviate from this (e.g. spirituality) were not examined.

## Supporting information

S1 Table Items of the right-wing extremism attitude scale. (DOCX)

S2 Table Items of the left-wing extremism attitude scale. (DOCX)

S3 Table Items of the Islamist extremism attitude scale. (DOCX)
