# Supplementary material for: Religion as an influencing factor of right-wing, left-wing and Islamist extremism. Findings of a Swiss youth study
Source: PLoS One. 2021 Jun 17;16(6):e0252851. doi: 10.1371/journal.pone.0252851 (PMC8211158; doi:10.1371/journal.pone.0252851)
Supplement: S1 Table — (DOCX) [file pone.0252851.s001.docx]

**S1 Table: Items of the right-wing extremism attitude scale**

|  | **mean** | **std. dev** |
| --- | --- | --- |
| Switzerland should belong only to the Swiss, who have already lived here for many generations. (nationalism) | 2.24 | 1.45 |
| The Swiss are innately superior to other nations. (social darwinism) | 2.07 | 1.36 |
| The Whites are world leaders, and rightly so. (racism) | 2.03 | 1.37 |
| If there are not enough jobs, the foreigners living in Switzerland should be sent back to their home country. (xenophobia) | 2.25 | 1.45 |
| Immigration into Switzerland should be forbidden for Muslims. (islamophobia) | 2.08 | 1.40 |
| Jews have too much power in Switzerland. (antisemitism) | 1.85 | 1.26 |
| I think it is okay to beat up foreigners in Switzerland because they are foreigners. (willingness to use violence against foreigners) | 1.39 | 0.93 |
| I think it is okay to verbally harass foreigners because they are foreigners. (willingness to use violence against foreigners) | 1.45 | 1.00 |
| I think it is okay if left-wing extremists, members of the Black Block, squatters, punks or the like are beaten up due to their political views. (willingness to use violence against left-wing extremists) | 1.55 | 1.06 |
| I think it is okay if the meeting points or houses of left-wing extremists, members of the Black Block, squatters, punks and the like are demolished. (willingness to use violence against left-wing extremists) | 1.61 | 1.12 |
