# Supplementary material for: Religion as an influencing factor of right-wing, left-wing and Islamist extremism. Findings of a Swiss youth study
Source: PLoS One. 2021 Jun 17;16(6):e0252851. doi: 10.1371/journal.pone.0252851 (PMC8211158; doi:10.1371/journal.pone.0252851)
Supplement: S2 Table — (DOCX) [file pone.0252851.s002.docx]

**S2 Table: Items of the left-wing extremism attitude scale**

|  | **mean** | **std. dev** |
| --- | --- | --- |
| We will really only be free, if the whole state is abolished. (communism/anarchy) | 1.97 | 1.29 |
| We do not need a state and no parties; we are the best at governing ourselves. (communism/anarchy) | 1.95 | 1.17 |
| The business leaders destroy the habitat of the simple people in the cities. (hostility towards capitalism) | 2.86 | 1.37 |
| The major companies around the world are responsible for poverty and hunger in the world. (hostility towards capitalism) | 3.39 | 1.43 |
| The police and state only protect the rights of the rich. (hostility towards the police and the state) | 2.51 | 1.34 |
| I think it is okay if the buildings or luxury cars of the major companies and business leaders around the world are damaged. (willingness to use violence against capitalists) | 1.68 | 1.13 |
| I think it is okay to use violence against the police (e.g. blows, kicks, rocks, pyros). (willingness to use violence against police officers) | 1.61 | 1.11 |
| I think it is okay to beat up right-wing extremists, xenophobes, fascists, Nazis or the like because of their political views. (willingness to use violence against right-wing extremists) | 1.86 | 1.31 |
| I think it is okay to demolish the meeting points of right-wing extremists, xenophobes, fascists, Nazis or the like. (willingness to use violence against right-wing extremists) | 2.01 | 1.43 |
