# Supplementary material for: Religion as an influencing factor of right-wing, left-wing and Islamist extremism. Findings of a Swiss youth study
Source: PLoS One. 2021 Jun 17;16(6):e0252851. doi: 10.1371/journal.pone.0252851 (PMC8211158; doi:10.1371/journal.pone.0252851)
Supplement: S3 Table — (DOCX) [file pone.0252851.s003.docx]

**S3 Table: Items of the Islamist extremism attitude scale**

|  | **mean** | **std. dev** |
| --- | --- | --- |
| The Swiss society must be reformed according to Islamic rules. (introduction of theocracy and Sharia) | 1.33 | 0.82 |
| The Islamic laws of Sharia, whereby adultery or homosexuality are severely punished, for example, are much better than then Swiss laws. (introduction of theocracy and Sharia) | 1.47 | 1.01 |
| Islam is the one true religion; all other religions are of lesser value. (superiority of Islam) | 1.30 | 0.87 |
| I am repulsed by the lifestyle of people in the western world (e.g. wearing expensive clothing, open sexuality). (devaluation of western societies) | 2.03 | 1.26 |
| Those who do not literally follow the rules of the Koran are not real Muslims. (hostility towards non-traditional Muslims) | 1.89 | 1.29 |
| Those who change the original Islam are betraying Islam. (hostility towards non-traditional Muslims) | 2.07 | 1.42 |
| The Swiss Christians are of less value than the Muslims here. (hostility towards Swiss) | 1.53 | 0.99 |
| I think it is okay to verbally harass people who do not belong to Islam as sceptics or the like. (willingness to use violence against non-Muslims) | 1.35 | 0.86 |
| I think it is okay if Muslims are physically punished because they did not abide by the religious rules. (willingness to use violence against non-Muslims) | 1.38 | 0.93 |
| I think it is okay if Muslims fight for their cause with violence and terrorist attacks. (Advocacy of terrorism/IS) | 1.42 | 1.06 |
| I think it is okay if young people go abroad to fight for the Islamic state or other Islamic groups. (Advocacy of terrorism/IS) | 1.50 | 1.08 |
